# Supplementary material for: Altered serum adiponectin and interleukin-8 levels are associated in the pathophysiology of major depressive disorder: A case-control study
Source: PLoS One. 2022 Nov 21;17(11):e0276619. doi: 10.1371/journal.pone.0276619 (PMC9678262; doi:10.1371/journal.pone.0276619)
Supplement: Attachment — Submitted filename: Response to Reviewers.docx [file pone.0276619.s001.docx]

Dear Editors and Reviewers,

Thank you for your letter and the reviewers' comments on our manuscript entitled "Altered serum adiponectin and IL-8 levels are associated in the pathophysiology of major depressive disorder: A case-control study" (Manuscript ID PONE-D-21-41053). All the comments were valuable and helpful to the revision and improvement of the manuscript. We have carefully studied the comments and made corrections, which we hope will merit your approval. We marked the revised portions using track changes. Our point-by-point answers to the reviewers’ comments appear at the end of this letter.

We earnestly appreciate the Editors'/Reviewers' work. We hope that after this revision, the paper will be deemed fit for publication. We would be glad to respond to any further questions and comments that you may have.

Once again, thank you very much for your comments and suggestions.

Best regards,

**Md. Rabiul Islam, PhD**

Associate Professor, Department of Pharmacy, University of Asia Pacific

74/A Green Road, Farmgate, Dhaka-1205, Bangladesh.

Email: robi.ayaan@gmail.com; Cell: +8801916031831; ORCID iD: https://orcid.org/0000-0003-2820-3144

**Point by point authors’ responses to the reviewers**

Manuscript ID PONE-D-21-41053

*Title: Altered serum adiponectin and IL-8 levels are associated in the pathophysiology of major depressive disorder: A case-control study*

**Reviewer #1**

The manuscript by Islam et al describes alterations of serum adiponectin and IL-8 levels in
MDD patients. They report that adiponectin was decreased and IL-8 level was increased in MDD patients. Moreover, they perform sex-specific analysis of serum adiponectin and IL-8 levels in MDD patients.

Overall, I feel that this manuscript was interesting, but confusing in part because the writing was not clear. In its present form, the manuscript is extremely difficult to follow and this markedly diminishes the reader's interest, I would strongly recommend the following revisions:

***Author responses***

*Thank you for your review and valuable observation. We appreciate your encouraging comments on our manuscript. The English language editing has been completed.*

**Comment 1:**

Have the manuscript proof read by a native English speaker. Some parts of the manuscript are better written than others e.g., the discussion is poor compared to the rest of the manuscript.

***Author responses***

*We have now edited the whole manuscript for correcting mistakes in both grammar and style by the person who is fluent in English. We think the revised manuscript is much better than previous one in terms of written English.*

**Comment 2:**

What the relation between serum adiponectin and IL-8 levels in MDD patients.

***Author responses***

*Thank you for your observation. We have now provided justification for combined assessment of adiponectin and IL-8 in depression in the revised manuscript. You will find this information in page 5, line 32-33; page 6, line 1-6.*

*The association between cytokine alterations and the pathogenesis of major depression is still obscure. Moreover, a balance between pro-inflammatory and anti-inflammatory effects is crucial in depression. Any imbalance in inflammatory markers might influence the pathophysiology of depression. IL-8 is prominently known for its pro-inflammatory effects and adiponectin provides anti-inflammatory effects. Based on the published evidence, we know little about the role of adiponectin and IL-8 in the development of depression. Therefore, the present study aimed to evaluate serum adiponectin and IL-8 in MDD through a case-control design.*

**Reviewer #2**

The manuscript submitted by Islam et al. aimed to compare serum adiponectin and interleukin-8 (IL-8) levels between individuals with major depressive disorder (MDD) and healthy subjects. The data showed an elevated IL-8 concentration and a reduced adiponectin level in patient with MDD compared to healthy control, and an association between both serum cytokines concentrations and the disease severity. The study also indicated that serum adiponectin and IL-8 levels exhibited good predictive performances and so, can be used as risk assessment factors in depression.
***Author responses***

*Thank you for your effort to review our manuscript.*

**Comment 1:**

My major criticism is that the goal of the study is not clearly described. Although the authors provide an exhaustive published literature on the topic, they failed to address the research question. More especially, they mentioned contradictory findings in some previous studies. Some of them have shown differences in serum adiponectin and IL-8 between individuals with MDD and healthy subjects while others did not. They also claimed that the role of adiponectin and IL-8 in the development of depression remains unclear and indicated that any suitable biomarkers for risk assessment of depression have not been established yet. So, what is the aim of the adiponectin and IL-8 assessment? Did the authors want to explain the contradictory findings, or investigate the role of these cytokines in depression or identify new biomarkers for MDD? The question posed should be more defined.
***Author responses***

*Thank you for your observation. We have now provided justification for combined assessment of adiponectin and IL-8 in depression in the revised manuscript. You will find this information in page 5, line 32-33; page 6, line 1-6.*

*The association between cytokine alterations and the pathogenesis of major depression is still obscure. Moreover, a balance between pro-inflammatory and anti-inflammatory effects is crucial in depression. Any imbalance in inflammatory markers might influence the pathophysiology of depression. IL-8 is prominently known for its pro-inflammatory effects and adiponectin provides anti-inflammatory effects. Based on the published evidence, we know little about the role of adiponectin and IL-8 in the development of depression. Therefore, the present study aimed to evaluate serum adiponectin and IL-8 in MDD through a case-control design.*

**Comment 2:**

Other comments
Originality. The authors did not state the novelty of the study and the manuscript provides minor advance over existing information.
***Author responses***

*Thank you for your opinion. We have now added some information based on what we already know about this topic and what is the present research’s contribution to the field. You will find this information in page 11, line 30-33; page 12, line 1-16.*

*The present study performed ROC curve analysis for altered adiponectin and IL-8 to estimate the predictive performances (Figure 3). Precision based on the area under the curve (AUC) in ROC analysis are as follows: <0.6 = not useful, 0.6-0.7 = poor, 0.7-0.8 = fair, 0.8-0.9 = good, and 0.9-1.0 = excellent [85]. Many past studies recognized the altered serum adiponectin and IL-8 in depression. But these alterations cannot be used as risk assessment fac-tors in depression due to the absence of their predictive performances [51,52,55,56,68,70]. According to the present study findings, the AUC of adiponectin and IL-8 were 0.895 and 0.806, respectively. Both the curve values were significant (p<0.001). Higher values of IL-8 and lower values of adiponectin were assigned as the disease state. Based on the ROC analysis results, both the markers showed good sensitivity, specificity, PPV, and NPV. Major de-pression multifactorial disease where many environmental, biological, and genetic factors are involved [86]. Therefore, the identification and definition of biological risk factors are vital for understanding the pathophysiology and management of depression. Evidence suggests that recovered MDD patients achieved a balance in the levels of pro-and anti-inflammatory markers after treatment [87]. However, the previous studies could not suggest possible interventions to resume the balance of altered adiponectin and IL-8 in depression based on their findings [51,55,68,70]. The present study findings suggest some therapeutic approaches for MDD patients. Mind-body therapies (MBTs), physical exercises, and nutritional food/supplementation as non-pharmacological interventional strategies that can decrease stress levels, correct altered biomarkers, and improve the quality of life of MDD patients.*

**Comment 3:**

Introduction. The authors provide numerous previous published data to introduce the topic of the study. However, the introduction section includes some details that are not required. For instance, I think that too much information on MDD prevalence and on pro-inflammatory changes in individuals with MDD are provided. Conversely, the contradictory findings on serum adiponectin and IL-8 should be described to better relate the study to previously published literature.
***Author responses***

*Thank you for your valuable suggestion. We have now revised the whole introduction section and reduced some less important information to make it more related to the present topic.*

**Comment 4:**

Methods. Overall, the research is technically sound. However, clinical characteristics that might represent biological subtypes of MDD and so might may influence the data, are lacking. The age of MDD onset, the speed of onset of depressive episodes or the medication status of the patients should be included. In addition, the authors claimed that participants with some criteria were excluded from the study. But what about patients with diabetes mellitus? This is an important point as this metabolic disorder may affect serum adiponectin levels. The ethics statement should be added in the method section.
***Author responses***

*Thank you for your suggestion.*

*It would be better if we could provide biological subtypes of MDD, age of MDD onset, the speed of onset of depressive episodes in the present article. We have added these as limitations of the present study (page 12, line 26-28).*

*We enrolled drug-free MDD patients for this study and excluded patients with diabetes mellitus. This information we mentioned in the method section (page 6, line 10, 22).*

*The ethics statement has been added in the last paragraph of method section (page 7, line 20-25).*

**Comment 5:**

Results.
The data are, for the most part, well set out. Figures 2 and 3 raise however some concerns:
- Figure 2. I do not understand the scatters plot graphs. What is this representation? According to the authors, Spearman’s correlation analysis have been performed to evaluate association among the parameters. If true, r (the coefficient correlation) and p (the statistical significance) values should be provided. What are the y values shown on graphs? r value should be between -1 and 1.
- Figure 3. I am not aware of the ROC curve analysis. I think that the findings would be more convincing if the authors explain both the significance of such analysis and how the conclusion may contribute to the study.
***Author responses***

*Thank you for your opinion.*

*We have now revised the Figure 2 by changing dimensions (x and y axes). The mean values in the fit lines are showing how adiponectin and IL-8 levels are changing with Ham-D score of MDD patients. We are seeing sex-specific changes through Figure 2 where we see* *female MDD patients showed lower serum levels at higher Ham-D scores and vice versa. Moreover, the rate of sex-specific changes of serum markers among MDD patients are higher among females than males.*

*We have now reported the r and p values of Spearman’s correlation analysis in the revised manuscript (page 8, line 21-26).*

*The y values in the earlier figure 2 were mean Ham-D scores of MDD patients by sex. These values are repetitive of Table 2, therefore, we omitted them from in the revised Figure 2.*

*We have now explained how the findings from ROC would contribute to the earlier findings and based on these findings how researchers can use present findings for identification of risk assessment markers for depression (page 9, line 5-13; page 11, line 30-33; page 12, line 1-16).*

**Comment 6:**

Other points:
As adipokines dysregulation is found in obese individuals, it would be more appropriate to consider the 3 BMI subgroups to compare serum adiponectin levels between MDD patients and healthy control. A meta-analysis suggested actually that studies involving samples with BMI ≥ 25 had lower adiponectin levels in subjects with MDD compared to HC (Cao et al., J Affect Disord, 2018).
***Author responses***

*Thank you for your valuable suggestion. We have now analyzed and presented serum markers among 3 BMI subgroups (Revised Table 2). However, we have seen that no changes of adiponectin levels among the samples with BMI ≥ 25 in subjects with MDD compared to HC.*

**Comment 7:**

The authors claimed that “the changes in serum adiponectin were prominent in females than in males” (p8). The data in table 2 do not support this interpretation. The mean serum adiponectin level was reduced by 43% in female MDD patient and by 41% in male patient.
***Author responses***

*Thank you for your opinion. For serum adiponectin levels were 27.72 ± 5.52 in male MDD patients and 47.25 ± 8.24 in male HCs (p=0.071), however, the levels were 33.51 ± 6.94 female MDD patients and 58.89 ± 7.07 in female HCs (p=0.019) (Table 2). From this data we can see that female participants showed significant changes whereas males do not. Also, you will see the sharp changes of serum adiponectin levels among females MDD patients than males in the revised Figure 2.*

**Comment 8:**

Please check for the unit used for serum IL-8. Usually, circulating cytokine levels are not so elevated.
***Author responses***

*Thank you for your observation. We have checked the unit of serum IL-8 and found same.*

**Comment 9:**

Conclusion
The authors should discuss the contribution of their findings to the previous published data. More especially, they should state whether their results might explain the contradictory studies.
Why the authors have considered only physical activity as a way to regulate serum cytokine levels while it is likely that antidepressant treatment might also change cytokines production?
The authors’ conclusion that “alterations of serum adiponectin and IL-8 may be the result of the pathophysiology of depression, and not the cause” is not supported by the data. Similarly, the data do not provide enough evidence to suggest the practical implications stated by the authors.

***Author responses***

*Thank you for your observation. We have now revised the conclusion.*

**Additional Editor Comments:**

The manuscript by Islam et al. describes alterations of serum adiponectin and IL-8 levels in MDD patients. They show a decrease in adiponectin level and an increase in IL-8 in MDD patients. A sex-specific analysis of serum adiponectin and IL-8 levels in MDD patients was also carried out.
Both reviewers find that the manuscript does not clearly state the purpose and impact of the study. In particular the relation between serum adiponectin and IL-8 levels in MDD patients should be explained.
Authors should clarify their conclusions and clearly explain what does their study bring over existing data.

Concerns raised by Reviewer 2 on Figures 2 and 3 have to be carefully addressed.

***Author responses***

*Thank you for your suggestion. We have revised the manuscript carefully following both reviewer’s comments and suggestions.*
