# Supplementary material for: Altered serum adiponectin and interleukin-8 levels are associated in the pathophysiology of major depressive disorder: A case-control study
Source: PLoS One. 2022 Nov 21;17(11):e0276619. doi: 10.1371/journal.pone.0276619 (PMC9678262; doi:10.1371/journal.pone.0276619)
Supplement: Attachment — Submitted filename: Response to Reviewers.docx [file pone.0276619.s002.docx]

Dear Editors and Reviewers,

Thank you again for your letter and the reviewers' comments on our manuscript entitled "Altered serum adiponectin and IL-8 levels are associated in the pathophysiology of major depressive disorder: A case-control study" (Manuscript ID PONE-D-21-41053.R1). All the comments were valuable and helpful to the revision and improvement of the manuscript. We have carefully studied the comments and made corrections, which we hope will merit your approval. We marked the revised portions using track changes. Our point-by-point answers to the reviewers’ comments appear at the end of this letter.

We earnestly appreciate the Editors'/Reviewers' work. We hope that after this revision, the paper will be deemed fit for publication. We would be glad to respond to any further questions and comments that you may have.

Once again, thank you very much for your comments and suggestions.

Best regards,

**Md. Rabiul Islam, PhD**

Associate Professor, Department of Pharmacy, University of Asia Pacific

74/A Green Road, Farmgate, Dhaka-1205, Bangladesh.

Email: robi.ayaan@gmail.com; Cell: +8801916031831; ORCID iD: https://orcid.org/0000-0003-2820-3144

**Point by point authors’ responses to the reviewers**

Manuscript ID PONE-D-21-41053.R1

*Title: Altered serum adiponectin and IL-8 levels are associated in the pathophysiology of major depressive disorder: A case-control study*

**Reviewer #1**

In this revision, the authors had addressed majority of my comments and I don't have any additional comments beyond what I raised in the initial review.

***Author responses***

*Thank you for your review and valuable opinion on the revised version of our manuscript.*

**Reviewer #2**

The reviewer would like to thank the authors for taking the recommendations into consideration and for their extensive point-by-point response to the initial review. Many efforts have been made to complete the paper with the clarifications and additional text. Overall, this is a well-written and thoughtful paper whose findings clearly address an important clinical issue for MDD patients. With that said, there are still minor issues that the authors should consider:

***Author responses***

*Thank you for your effort to review our manuscript and encouraging comments.*

**Comment 1:**

• Thank you for adding data from Spearman’s correlation analysis. However, they do not provide significant conclusions and the interpretation is not correct. A so low r value found for serum adiponectin levels means that this cytokine may not be associated with MDD. In addition, the authors claimed that IL-8 levels showed opposite results compared to adiponectin whereas both r value are minus number. I think that Spearman’s correlation analysis should be discarded because they provided confusing findings over data from scatter plot graphs (figure 2).

***Author responses***

*Thank you for your observation and valuable suggestion. We also believe that Spearman’s correlation analysis does not add significant value to the present findings, at the same time creating some confusion. Therefore, we discarded this analysis from the method, results and discussion sections following the reviewer’s suggestion.*

**Comment 2:**

• Although the authors have checked the unit used for serum IL-8 levels, I doubt whether they reach so high values. I did not get any information on the ELISA assay kit (I can’t get hold of the manufacturer anywhere), but serum IL-8 levels were shown to be ranged from 1 to 1000 pg/ml (An Z et al. Cell cycle, 2019, vol.18, p2928; - Amoras EDSG et al. Biomolecules, 2021 vol. 11 p1664 – Heldt S et al. Mycoses, 2017, vol.60, p818 – Sinegar GA et al. Acta Med Indones, 2015, vol.47, p120). Serum IL-8 levels around µg/ml are therefore unlikely.

***Author responses***

*Thank you for your valuable observation. The unit of our calculated serum adiponectin and IL-8 levels was also pg/ml (Ref:* [*https://www.bosterbio.com/datasheet?sku=EK0413*](https://www.bosterbio.com/datasheet?sku=EK0413)*; https://www.bosterbio.com/datasheet?sku=EK0595). We wrote the unit as μg/ml by mistake for both the markers and we have now corrected the unit throughout the manuscript and other places where applicable (manuscript, Table 2, Figure 1 & 2). We are showing our apology for this mistake.*

**Comment 3:**

• Some affirmations are not correct. At the beginning of the paragraph on the “Potential limitations of the study”, the authors cannot claim that “their approach aimed to be comprehensive” as it is a descriptive study. Similarly, they cannot state that “the altered serum adiponectin and IL-8 levels can be used as early risk assessment tools for depression” because the age of MDD onset remains unknown.

***Author responses***

*Thank you for your valuable suggestion. We have now corrected them accordingly (page 12, line 5-6; page 13, lines 10-11).*
